# Long-term survival of women with basal-like ductal carcinoma in situ of the breast: a population-based cohort study

**DOI:** 10.1186/1471-2407-10-653

**Published:** 2010-11-30

**Authors:** Wenjing Zhou, Karin Jirström, Christine Johansson, Rose-Marie Amini, Carl Blomqvist, Olorunsola Agbaje, Fredrik Wärnberg

**Affiliations:** 1 Department of Surgical Science, Uppsala University, Uppsala, SE-75105, Sweden; 2 Department of Laboratory Medicine, Lund University, Lund, Sweden; 3 Department of Genetics and Pathology, Uppsala University, Uppsala, Sweden; 4 Department of Oncology, Helsinki University Central Hospital, Finland; 5 School of Medicine, Division of Cancer Studies, King's College, London, UK

## Abstract

**Background:**

Microarray gene-profiling of invasive breast cancer has identified different subtypes including luminal A, luminal B, HER2-overexpressing and basal-like groups. Basal-like invasive breast cancer is associated with a worse prognosis. However, the prognosis of basal-like ductal carcinoma *in situ (*DCIS) is still unknown. Our aim was to study the prognosis of basal-like DCIS in a large population-based cohort.

**Methods:**

All 458 women with a primary DCIS diagnosed between 1986 and 2004, in Uppland and Västmanland, Sweden were included. TMA blocks were constructed. To classify the DCIS tumors, we used immunohistochemical (IHC) markers (estrogen-, progesterone-, HER2, cytokeratin 5/6 and epidermal growth factor receptor) as a surrogate for the gene expression profiling. The association with prognosis was examined for basal-like DCIS and other subtypes using Kaplan-Meier survival analyses and Cox proportional hazards regression models.

**Results:**

IHC data were complete for 392 women. Thirty-two were basal-like (8.2%), 351 were luminal or HER2-positive (89.5%) and 9 unclassified (2.3%). Seventy-six women had a local recurrence of which 34 were invasive. Another 3 women had general metastases as first event. Basal-like DCIS showed a higher risk of local recurrence and invasive recurrence 1.8 (Confidence interval (CI) 95%, 0.8-4.2) and 1.9 (0.7-5.1), respectively. However, the difference was not statistically significant. Also, no statistically significant increased risk was seen for triple-negative or high grade DCIS.

**Conclusions:**

Basal-like DCIS showed about a doubled, however not statistically significant risk for local recurrence and developing invasive cancer compared with the other molecular subtypes. Molecular subtyping was a better prognostic parameter than histopathological grade.

## Background

Increased use of screening mammography, beginning in the early 1980 s, has resulted in a dramatic increase in detection of ductal carcinoma *in situ *(DCIS). DCIS is a pre-invasive disease with a clinically and molecularly heterogeneous presentation that poses a major challenge in both diagnosis and treatment [1-3]. A clinically accepted classification system predicting prognosis is still missing.

Recently, the idea of using molecular subtyping to predict the prognosis of invasive breast cancer has been widely accepted [4-8]. The introduction of high-throughput DNA microarray technologies marked an entirely new era of genome-wide approaches to predict prognosis and outcome [9,10]. According to similarities in gene expression characteristics, Perou et al., [11] creatively classified breast cancer into five subtypes: luminal A, luminal B, basal like, HER2-positive and normal-like. Molecular subtypes have been shown to have prognostic and treatment predictive value. Among those, the basal-like tumors are characterized as expressing genes related to myoepithelial cells [12]. Follow-up studies have shown that basal-like cancer is associated with a higher risk of disease recurrence, distant metastasis and shorter survival. The poorer outcome of patients with basal-like tumors has been shown in different patient populations [13-17]. However, the prognosis of basal-like DCIS still unknown.

In this study, our aim was to investigate the prognosis of basal-like DCIS compared to other subtypes in a large population-based cohort. To classify the DCIS tumors, we used immunohistochemical (IHC) markers that have previously been used as a surrogate marker for the gene expression profiles [16-21]. Livasy et al., have earlier shown that IHC can be used for the identification of basal like DCIS [18]. We identified the subtypes of DCIS in tumors from a cohort of 458 women with tumor material assembled in tissue micro arrays (TMA) [22]. To our knowledge, this study is the first to examine the prognosis of DCIS in relation to different molecular subtypes defined by IHC.

## Methods

### Patients

We recruited all 458 women who were diagnosed with a primary DCIS between 1986 and 2004 in Uppland and Västmanland, Sweden.

### TMA construction

H&E sections from all eligible cases were reviewed to select the area from which the cores for the TMAs would be taken. Prior to the TMA construction, all primary DCIS cases were histopathologically re-evaluated by one pathologist (KJ). Two cores of 1.0 mm in diameter were mounted into the recipient TMA blocks using a manual arraying device (MTA-1, Beecher Inc, WI, USA). The concordance of IHC staining between original whole section slides and TMA-slides and between biopsies from the same lesion in DCIS has earlier been evaluated in a subset of the cohort [23,24]. In total, ten TMA blocks were constructed from 458 cancer samples.

### IHC and silver-enhanced in situ hybridization (SISH)

We performed IHC for estrogen receptor (ER), progesterone receptor (PR), human epidermal growth factor receptor 2 (HER2), cytokeratin 5/6 (CK5/6) and epidermal growth factor receptor (EGFR) on 4 μm paraffin sections cut from the TMAs. Immunostains for each marker were performed on a Dako Autostainer (Dako Corporation). IHC was conducted according to established protocols. Appropriate positive and negative controls were included in all staining runs.

HER2 SISH was performed on an automated instrument, Ventana Benchmark (Ventana Medical Systems, Tucson, AZ), as per the manufacturer's protocols for the INFORM HER2 DNA probe and chromosome 17 probes. Testing for the HER2 gene and chromosome 17 was performed on sequential sections. Both probes are labeled with dinitrophenol. Denaturation occurred on the instrument with enzyme digestion in protease 3 for 8 minutes. The detection system used a multimer labeled with goat antirabbit antibody horseradish peroxidase as the linking step. Visualization occurred with the sequential addition of silver acetate as the source of ionic silver, hydroquinone, and hydrogen peroxide to give a black metallic silver precipitate at the probe site. Counterstaining was performed with hematoxylin II on the instrument. The time taken for the complete run is 6.5 hours. Both HER2 and chromosome 17 detection were performed on the same slide run. Gene amplification was assessed using the American Society of Clinical Oncology/College of American Pathologists guideline and Australian HER2 Advisory Board criteria for single HER2 probe testing (diploid, 1 to 2.5 copies/nucleus; polysomy >2.5 to 4 copies/nucleus; equivocal, >4 to 6 copies/nucleus; low-level amplification, >6 to 10 copies/nucleus; and high-level amplification >10 copies/nucleus) and for dual HER2/CHR17 probe testing (nonamplified ratio <1.8; equivocal ratio, 1.8 to 2.2; gene amplification, >2.2). The status of HER2 protein expression was assessed relying on SISH. For those cases on which SISH failed, the expression was based on IHC.

### Scoring and classification

Stained TMA slides were scanned (ScanScope^® ^XT, Aperio, USA) for evaluation of expression of ER, PR, HER2, CK5/6 and EGFR by ImageScope (Aperio, USA). Tumor cells that showed nuclear staining for ER or PR (> 10% of tumor cell nuclei staining) were considered ER or PR-positive respectively. Using the HerceptTest™classification system, tumors were considered HER2 positive if the score was 3+. Cases were considered CK 5/6-positive or EGFR-positive if any cytoplasmic and/or membranous staining was detected in the tumor cells, even if focal. These latter IHC criteria are similar to those previously used for scoring these markers in invasive breast cancers [18,22,25,26]. If only one core included enough tumor tissue this was used for classification but at least 200 cells had to be present. Each tumor marker was scored by one single observer; ER, PR and HER2 by WZ, EGFR and CK5/6 by CJ and SISH by RMA.

For this study, the IHC subtype definitions used were the refined definitions used in the analysis of invasive breast cancer: basal-like (ER-, HER2-, EGFR+ and/or cytokeratin 5/6+) [17]. Cases classified as negative for all the above markers were defined as unclassified is this study. Cases with missing data, due to lack of tumor tissue in the TMAs, were excluded from the further analyses. In the survival analyses we compared the basal-like group with all other DCIS together.

### Follow-up and Study End Points

Follow-up started on the day of diagnosis and concluded on the date the patient was last observed or the date of death. The database was frozen for the statistical analyses based on follow-up through 30th April 2008. Complete follow-up was achieved for all 458 patients.

For this study, the two primary end points were local recurrence and invasive- or general recurrence. Local recurrence was defined as any ipsilateral recurrence (*in situ *or invasive). In addition, we defined "invasive- or general recurrence" as occurrence of an invasive ipsilateral recurrence, general metastasis or breast cancer-specific death, whichever occurred first. All women with an invasive local recurrence were included as cases regarding both endpoints. The endpoint "invasive- or general recurrence" was constructed to find patients with a biologically more aggressive disease. Contralateral breast tumors were considered as new primary breast cancers.

### Statistic analyses

Baseline characteristics among patients with different molecular subtypes were compared by Chi-square for categorical variables or analysis of variance for continuous variables. Survival and probabilities of local recurrence and invasive disease among patients with different molecular subtypes were first compared by the Kaplan-Meier method. Cox proportional hazards regression models were used to calculate hazard ratios (HRs) with 95% confidence intervals (CIs), with adjustment for age in the univariate analysis and further, histological grade, type of surgery, tumor size, detection mode and postoperative radiotherapy in the multivariate analysis. Kaplan-Meier curves and Cox proportional hazard models were used to assess the association between molecular subgroup and progression of breast cancer. Data were analyzed using the SAS (SAS Institute, Cary, NC) and R softwares.

This study was approved by the Ethics Committee at Uppsala University Hospital (Dnr 2005: 118). In this retrospective cohort study we did not have to get an informed consent from the women according to the ethical approval, Dnr 170/95 and 99/422.

## Results

### 1. Baseline Characteristics

Baseline characteristics according to the molecular subtype of 392 eligible study participants (66 women did not get enough IHC information) are presented in Table 1. The median follow-up was 122 months (range 3-130). Among the 392 women, 32 (8.2%) were classified as basal-like, 351 (89.5%) as luminal or HER2-positive, and 9 (2.3%) were unclassified. The median age at diagnose was 58 years (range 30-90 years). All basic characteristics, including age, tumor size and grade, were evenly distributed between the basal-like and non basal-like tumors. No women received antiestrogen or chemotherapy after primary surgery. Type of surgery, mastectomy vs. breast conserving surgery (BCS) and postoperative radiotherapy were decided according to local tradition. BCS in Sweden means removing a sector of the breast parenchyma in up to a quadrant of the breast, aiming for a 10 mm free margin. The breast parenchyma is removed from behind the areola margin to the periphery of the gland. Postoperative radiotherapy was given to 140 of 298 women undergoing BCS. It was generally given at 25 occasions, up to 50 Grey. A small part of these women were included in a Swedish study randomizing between radiotherapy or not after BCS for a primary DCIS [27]. Others were given radiotherapy based on tumor size and histopathological grade. However, at that time, no criteria for radiotherapy regarding size or grade were given in the Swedish National Guidelines.

**Table 1:** Characteristics of DCIS molecular subgroups, by immunohistochemistry in 392 women with a primary DCIS

Characteristics	Basal-like (n = 32, 8.2%)	Non basal-like (n = 360, 91.8%)	*P*
Age at entry, mean ± SD (n = 392)	56.7 ± 12.6	57.8 ± 11.5	0.6
Tumor size, number (%) (n = 352)			0.84
≤ 15 m	16 (59.3)	175 (53.9)	
> 15 mm	8 (29.6)	104 (32.0)	
Multifocal	3 (11.1)	46 (14.2)	
Tumor grade*, number (%) (n = 388)			0.11
I	1 (3.1)	29 (8.2)	
II	11 (34.4)	171 (48.0)	
III	20 (62.5)	156 (43.8)	
Detection mode, number (%) (n = 391)			0.3
Screening	22 (68.8)	276 (76.9)	
Clinically	10 (31.3)	83 (23.1)	
Type of surgery, number (%) (n = 392)			0.61
Breast Conserving Surgery	26 (81.3)	272 (75.6)	
Mastectomy	6 (18.8)	88 (24.4)	
Postoperative radiotherapy, number (%)(n = 392)			0.72
Yes	10 (31.3)	130 (36.1)	
No	22 (68.8)	230 (63.9)	

* Tumor grade: DCIS were classified according to the European Organization for Research and Treatment of Cancer (EORTC) system.

### 2. Survival Outcomes

#### 2.1 Local-recurrence

**Figure 1:**
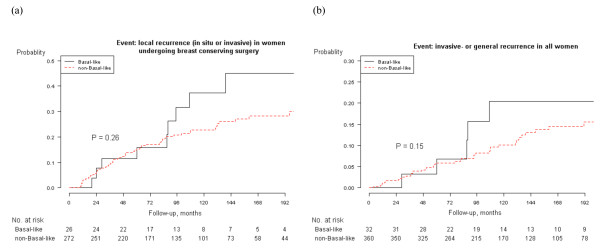
**Kaplan-Meier analyses of local recurrence and invasive- and general recurrence by DCIS molecular subgroup by immunohistochemistry**. (a) Local recurrence (in situ or invasive) in women with a primary DCIS, undergoing breast-conserving surgery (BCS), by molecular subtype. (b) Invasive- or general recurrence in all women with a primary DCIS by molecular subtype.

Of the 76 women who had a local recurrence, 42 had an *in situ *recurrence and 34 an invasive recurrence. Median follow-up to local recurrence was 97.5 months (range 3-255). In both the univariate and multivariate models, basal-like DCIS showed a higher risk of local recurrence compared with non basal-like DCIS, hazard ratio (HR), 1.7 (CI 95%: 0.8-3.8) and 1.8 (0.8-4.2) respectively (Table 2). However, the difference was not statistically significant. When only looking at women who had BCS, the risk for basal-like DCIS was equally higher (Table 2). Kaplan-Meier analyses were also made for all women and those undergoing BCS separately. The analyses showed a higher risk for basal-like DCIS compared to the non basal-like group but it was not statistically significant. The p-value was 0.08 for all women and 0.26 for women undergoing BCS (Figure 1a).

**Table 2:** Cox regression analyses by molecular subgroup, by immunohistochemistry

	All (n = 392)		Breast Conserving Surgery (n = 298)	
		
	Univariate HR (95% CI)	Adjusted* HR (95% CI)	Univariate HR (95% CI)	Adjusted* HR (95% CI)
	**Event: local recurrence (n = 76)**			
	
Gene-expression classification				
Non basal-like (n = 360)	1 (Reference)	1	1	1
Basal-like (n = 32)	1.7 (0.8-3.8)	1.9 (0.8-4.2)	1.6 (0.7-3.6)	1.8 (0.7-4.2)
Triple-negative classification				
Non triple-negative (n = 377)	1 (Reference)	1	1	1
Triple-negative (n = 32)	1.1 (0.4-2.7)	1.1 (0.4-2.9)	1.0 (0.4-2.7)	1.0 (0.3-2.9)
Tumor grade				
I (n = 30)	1 (Reference)	1	1	1
II (n = 182)	1.2 (0.4-3.4)	1.4 (0.5-4.0)	1.3 (0.5-3.9)	1.4 (0.5-3.9)
III (n = 176)	1.0 (0.3-2.9)	1.3 (0.5-3.9)	1.4 (0.5-4.0)	1.4 (0.5-4.2)
Postoperation radiotherapy				
No (n = 252)	1 (Reference)	1	1	1
Yes (n = 140)	0.9 (0.5-1.5)	0.7 (0.4-1.3)	0.8 (0.4-1.4)	0.7 (0.4-1.4)
				
	**Event: invasive- and general recurrence (n = 47)**			
	
Gene-expression classification				
Non basal-like (n = 360)	1 (Reference)	1	1	1
Basal-like (n = 32)	2.0 (0.8-5.0)	1.9 (0.7-5.1)	2.2 (0.8-5.6)	2.3 (0.8-6.1)
Triple-negative classification				
Non triple-negative (n = 377)	1 (Reference)	1	1	1
Triple-negative (n = 32)	1.4 (0.5-4.0)	1.6 (0.6-4.8)	1.7 (0.6-4.9)	2.1 (0.7-6.3)
Tumor grade				
I (n = 30)	1 (Reference)	1	1	1
II (n = 182)	1.0 (0.3-3.3)	1.1 (0.3-3.8)	1.1 (0.3-3.8)	1.1 (0.3-3.8)
III (n = 176)	0.7 (0.2-2.4)	1.1 (0.3-3.9)	0.9 (0.2-3.1)	1.0 (0.3-3.9)
Postoperation radiotherapy				
No (n = 252)	1 (Reference)	1	1	1
Yes (n = 140)	0.8 (0.4-1.6)	0.7 (0.3-1.5)	0.7 (0.3-1.4)	0.7 (0.3-1.6)

#### 2.2 Invasive- or general recurrence

Forty-seven women developed invasive disease. Forty-four had an invasive local recurrence (of which 10 first had an *in situ *local recurrence and then an invasive local recurrence). Three women had general metastases as the first event. Eight died from breast cancer, but all of these had had a local recurrence or general metastases before death. Median follow-up to an invasive- or general recurrence was 111.5 months (range 3-255). Basal-like DCIS showed a higher risk of invasive- or general recurrence compared with non basal-like DCIS, HR, 2.0 (CI 95%: 0.8-5.0) and 1.9 (0.7-5.1) respectively (Table 2). However, the difference was not statistically significant. When only looking at women who had BCS, the risk for basal-like DCIS was also about doubled, but the risk was not statistically significant higher (Table 2). Kaplan-Meier analyses also showed a non-statistically higher risk for invasive- or general recurrence for the basal-like DCIS (p = 0.15) as seen in Figure 1b and the p-value for women undergoing BCS was 0.16.

#### 2.3 Triple-negative group vs. non triple-negative group

We also assessed the prognostic implications of triple-negative (ER-, PR- and HER2-negative) vs. non triple-negative DCIS. Of all 458 women, 409 could be classified. Thirty two were classified as triple-negative and 377 as not. Triple-negative tumors had a HR of 1.1 (95% CI, 0.4-2.7) and 1.1 (0.4-2.9) for local recurrence compared with the non triple-negative in the univariate and multivariate analyses, respectively. The adjusted HR for an invasive- or general recurrence in the triple-negative group compared with women in non triple-negative was 1.4 (0.5-4.0) and 1.6 (0.6-4.8).

**Figure 2:**
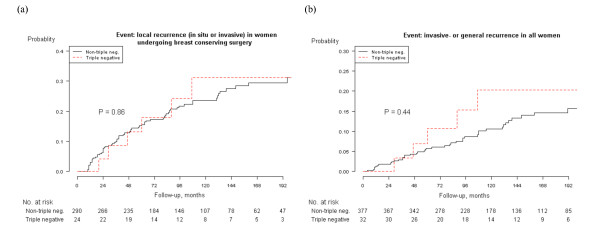
**Kaplan-Meier analysis of local recurrence and invasive- and general recurrence by triple-negative and non triple-negative DCIS**. (a) Local recurrence (BCS), triple negative DCIS vs. non-triple negative DCIS. (b) Invasive- or general recurrence in all women, triple negative DCIS vs. non-triple negative DCIS.

Kaplan-Meier analyses showed no statistically significantly higher risk for local recurrence or invasive- or general recurrence for the triple-negative DCIS, neither in the subset of women undergoing BCS (p = 0.86 and 0.25 respectively) nor including all women (p = 0.67 and 0.44 respectively). (Table 2 and Figure 2a-b).

Of the 392 women with complete IHC data, 32 were classified as basal-like and 32 as triple-negative. However, only 24 women were classified as both basal-like and triple-negative. These subgroups were too small to do any statistical analyses on but during the follow-up, the 8 women who were basal-like and non triple-negative had four invasive- or general recurrences and the 8 women who were triple-negative and non basal-like only had one invasive recurrence.

## Discussion

We have conducted a population-based study of primary DCIS to assess the association between an IHC surrogate basal-like DCIS genotype and prognosis. Our results showed that basal-like DCIS had a higher risk of local recurrence and a higher risk of developing invasive cancer compared to other DCIS. The risk was almost doubled but this was not statistically significant.

In our study, we defined basal-like DCIS as those that were ER-negative, HER2-negative, but positive for either CK 5/6 or EGFR. Nielsen et al [19], who proposed these criterions in 2004 for invasive breast cancer, found that defining basal-like breast cancer in this way resulted in 76% sensitivity and 100% specificity, when the gene expression signature was used as gold standard. Abd El-Rehim et al., [28] studied over 1000 cases of invasive breast cancer with 26 IHC markers and identified 6 clusters of tumors. Five of these clusters roughly corresponded to the 5 subtypes identified earlier; the remaining group consisted of only 4 cases. Livasy et al., [18] then verified and confirmed the criteria in DCIS tumors. Today, IHC is increasingly used as a surrogate for molecular gene profiling since it allows classification of tumors at an affordable costs and in the absence of fresh tissue speciments [29]. Moreover, these four subgroups have distinguishing features closely associated with subtypes defined by gene expression profiling, including distinct clinical outcomes [30].

A possible cause of subtype misclassification is the HER2 status based on IHC scoring. We assessed the status of HER2 by SISH and used IHC only in those cases where SISH failed. SISH results are more reliable [31,32] especially for the borderline cases scored as 2+ by IHC. By using both methods we should have minimized the risk of subtype misclassification.

Our results indicated that the molecular subtypes of DCIS were not related to basic characteristics as histopathological grade and tumor size. However, many potential prognostic factors may interact with the choice of adjuvant therapy [29] and we therefore adjusted for adjuvant treatment and type of surgery in the analyses. In this study, no women received chemotherapy or anti-estrogen. One hundred and forty received postoperative radiotherapy. The percentage of patients who received postoperative radiotherapy was fairly evenly distributed among the groups with different subtype of DCIS. The type of surgery was also evenly distributed.

Basal-like invasive breast cancer is an entity that is largely characterized by positive expression of basal CKs and other genes that are characteristic of basal-like cells and by high proliferative activity. Basal-like invasive breast cancer is considered to be associated with poor prognosis [13] and this supposition has been proved in many studies [4,5,13,17,33,34]. In our study, we found about a doubled risk for local recurrence and invasive- or general recurrences for basal-like DCIS. The higher risk was not statistically significant but, on the other hand, it was consistent in the univariate, multivariate and Kaplan-Meier analyses for all patients and for the subgroup of patients with BCS. Although we have conducted a population-based study with long follow-up, we were not able to show a statistically significantly poorer prognosis for the basal-like DCIS. Like in invasive breast cancer, the basal-like subgroup is small and this makes the statistical power low and a conclusive study would have to include a much larger number of patients. Today, we take nuclear grade, size, necrosis and margins into consideration when designing adjuvant treatment after BCS of DCIS according to the Swedish National Guidelines from the Swedish Breast Cancer Group [35]. In this study, grade did not relate to prognosis (see Table 2) and molecular subtyping by IHC might indeed be more useful.

We also investigated the prognosis of triple-negative DCIS. In invasive cancer, triple-negative tumors have been shown to have a worse prognosis [36-38]. We observed a slightly elevated risk for developing invasive disease, but this was not statistically significant. In some studies, triple-negative and basal-like DCIS cases have been considered to have a fairly similar prognosis. In our study, there was a considerable miss-match between these cases as already described in invasive breast cancer by others [21,39,40].

## Conclusions

In conclusion, basal-like DCIS showed about a doubled, however not statistically significant risk for local recurrence and developing invasive cancer compared with the other molecular subtypes. In this study, molecular subtyping was a better prognostic parameter than histopathological grade.

## List of abbreviations

DCIS: ductal carcinoma *in situ*; IHC: immunohistochemistry; CI: Confidence interval; HR: hazard ratio; TMA: tissue microarrays; ER: oestrogen receptor; PR: progesterone receptor; HER2: human epidermal growth factor receptor 2; CK5/6: cytokeratin 5/6; EGFR: epidermal growth factor receptor.

## Competing interests

The authors declare that they have no competing interests.

## Authors' contributions

WZ was responsible for data analyses, manuscript preparation and editing. KJ performed IHC and SISH stainings from the TMAs, and helped to provide expertise in breast cancer pathology. WZ, JC and RMA were involved in pathology review, scoring of stains and contributed substantially to manuscript editing. CB helped with the interpretation of the results and with drafting the manuscript. OA helped to provide expertise in data analyses. FW designed the overall study, compiled and curated the datasets, coordinated the study and helped to draft and finalize the manuscript. All authors read and approved the final manuscript.

## Pre-publication history

The pre-publication history for this paper can be accessed here:

http://www.biomedcentral.com/1471-2407/10/653/prepub

## References

[B1] Turashvili G Bouchal J Baumforth K Wei W Dziechciarkova M Ehrmann J Klein J Fridman E Skarda J Srovnal J Novel markers for differentiation of lobular and ductal invasive breast carcinomas by laser microdissection and microarray analysis BMC cancer 2007 7 55 10.1186/1471-2407-7-55 PMC1852112 17389037

[B2] Tsikitis VL Chung MA Biology of ductal carcinoma in situ classification based on biologic potential American journal of clinical oncology 2006 29 3 305 310 10.1097/01.coc.0000198740.33617.2f 16755185

[B3] Namba R Maglione JE Davis RR Baron CA Liu S Carmack CE Young LJ Borowsky AD Cardiff RD Gregg JP Heterogeneity of mammary lesions represent molecular differences BMC cancer 2006 6 275 10.1186/1471-2407-6-275 17147824 PMC1762020

[B4] Zhao J Liu H Wang M Gu L Guo X Gu F Fu L Characteristics and prognosis for molecular breast cancer subtypes in Chinese women J Surg Oncol 2009 100 2 89 94 10.1002/jso.21307 19544363

[B5] Spitale A Mazzola P Soldini D Mazzucchelli L Bordoni A Breast cancer classification according to immunohistochemical markers: clinicopathologic features and short-term survival analysis in a population-based study from the South of Switzerland Ann Oncol 2009 20 4 628 635 10.1093/annonc/mdn675 19074747

[B6] Nofech-Mozes S Trudeau M Kahn HK Dent R Rawlinson E Sun P Narod SA Hanna WM Patterns of recurrence in the basal and non-basal subtypes of triple-negative breast cancers Breast Cancer Res Treat 2009 19189211 10.1007/s10549-008-0295-8

[B7] Lund MJ Trivers KF Porter PL Coates RJ Leyland-Jones B Brawley OW Flagg EW O'Regan RM Gabram SG Eley JW Race and triple negative threats to breast cancer survival: a population-based study in Atlanta, GA Breast Cancer Res Treat 2009 113 2 357 370 10.1007/s10549-008-9926-3 18324472

[B8] Wirapati P Sotiriou C Kunkel S Farmer P Pradervand S Haibe-Kains B Desmedt C Ignatiadis M Sengstag T Schutz F Meta-analysis of gene expression profiles in breast cancer: toward a unified understanding of breast cancer subtyping and prognosis signatures Breast Cancer Res 2008 10 4 R65. 10.1186/bcr2124 18662380 PMC2575538

[B9] Schena M Shalon D Davis RW Brown PO Quantitative monitoring of gene expression patterns with a complementary DNA microarray Science 1995 270 5235 467 470 10.1126/science.270.5235.467 7569999

[B10] Pease AC Solas D Sullivan EJ Cronin MT Holmes CP Fodor SP Light-generated oligonucleotide arrays for rapid DNA sequence analysis Proceedings of the National Academy of Sciences of the United States of America 1994 91 11 5022 5026 10.1073/pnas.91.11.5022 8197176 PMC43922

[B11] Perou CM Sorlie T Eisen MB van de Rijn M Jeffrey SS Rees CA Pollack JR Ross DT Johnsen H Akslen LA Molecular portraits of human breast tumours Nature 2000 406 6797 747 752 10.1038/35021093 10963602

[B12] Sorlie T Wang Y Xiao C Johnsen H Naume B Samaha RR Borresen-Dale AL Distinct molecular mechanisms underlying clinically relevant subtypes of breast cancer: gene expression analyses across three different platforms BMC Genomics 2006 7 127 10.1186/1471-2164-7-127 16729877 PMC1489944

[B13] Sorlie T Perou CM Tibshirani R Aas T Geisler S Johnsen H Hastie T Eisen MB van de Rijn M Jeffrey SS Gene expression patterns of breast carcinomas distinguish tumor subclasses with clinical implications Proceedings of the National Academy of Sciences of the United States of America 2001 98 19 10869 10874 10.1073/pnas.191367098 11553815 PMC58566

[B14] Sorlie T Tibshirani R Parker J Hastie T Marron JS Nobel A Deng S Johnsen H Pesich R Geisler S Repeated observation of breast tumor subtypes in independent gene expression data sets Proceedings of the National Academy of Sciences of the United States of America 2003 100 14 8418 8423 10.1073/pnas.0932692100 12829800 PMC166244

[B15] Sotiriou C Neo SY McShane LM Korn EL Long PM Jazaeri A Martiat P Fox SB Harris AL Liu ET Breast cancer classification and prognosis based on gene expression profiles from a population-based study Proceedings of the National Academy of Sciences of the United States of America 2003 100 18 10393 10398 10.1073/pnas.1732912100 12917485 PMC193572

[B16] Potemski P Kusinska R Watala C Pluciennik E Bednarek AK Kordek R Prognostic relevance of basal cytokeratin expression in operable breast cancer Oncology 2005 69 6 478 485 10.1159/000090986 16410686

[B17] Carey LA Perou CM Livasy CA Dressler LG Cowan D Conway K Karaca G Troester MA Tse CK Edmiston S Race, breast cancer subtypes, and survival in the Carolina Breast Cancer Study JAMA 2006 295 21 2492 2502 10.1001/jama.295.21.2492 16757721

[B18] Livasy CA Perou CM Karaca G Cowan DW Maia D Jackson S Tse CK Nyante S Millikan RC Identification of a basal-like subtype of breast ductal carcinoma in situ Hum Pathol 2007 38 2 197 204 10.1016/j.humpath.2006.08.017 17234468

[B19] Nielsen TO Hsu FD Jensen K Cheang M Karaca G Hu Z Hernandez-Boussard T Livasy C Cowan D Dressler L Immunohistochemical and clinical characterization of the basal-like subtype of invasive breast carcinoma Clin Cancer Res 2004 10 16 5367 5374 10.1158/1078-0432.CCR-04-0220 15328174

[B20] Banerjee S Reis-Filho JS Ashley S Steele D Ashworth A Lakhani SR Smith IE Basal-like breast carcinomas: clinical outcome and response to chemotherapy J Clin Pathol 2006 59 7 729 735 10.1136/jcp.2005.033043 16556664 PMC1860434

[B21] Cheang MC Voduc D Bajdik C Leung S McKinney S Chia SK Perou CM Nielsen TO Basal-like breast cancer defined by five biomarkers has superior prognostic value than triple-negative phenotype Clin Cancer Res 2008 14 5 1368 1376 10.1158/1078-0432.CCR-07-1658 18316557

[B22] Livasy CA Karaca G Nanda R Tretiakova MS Olopade OI Moore DT Perou CM Phenotypic evaluation of the basal-like subtype of invasive breast carcinoma Mod Pathol 2006 19 2 264 271 10.1038/modpathol.3800528 16341146

[B23] Warnberg F Amini RM Goldman M Jirstrom K Quality aspects of the tissue microarray technique in a population-based cohort with ductal carcinoma in situ of the breast Histopathology 2008 53 6 642 649 10.1111/j.1365-2559.2008.03156.x 19076680

[B24] Jirstrom K Ringberg A Ferno M Anagnostaki L Landberg G Tissue microarray analyses of G1/S-regulatory proteins in ductal carcinoma in situ of the breast indicate that low cyclin D1 is associated with local recurrence Br J Cancer 2003 89 10 1920 1926 10.1038/sj.bjc.6601398 14612904 PMC2394433

[B25] Jumppanen M Gruvberger-Saal S Kauraniemi P Tanner M Bendahl PO Lundin M Krogh M Kataja P Borg A Ferno M Basal-like phenotype is not associated with patient survival in estrogen-receptor-negative breast cancers Breast Cancer Res 2007 9 1 R16. 10.1186/bcr1649 17263897 PMC1851391

[B26] Ihemelandu CU Leffall LD Dewitty RL Naab TJ Mezghebe HM Makambi KH Adams-Campbell L Frederick WA Molecular breast cancer subtypes in premenopausal African-American women, tumor biologic factors and clinical outcome Ann Surg Oncol 2007 14 10 2994 3003 10.1245/s10434-007-9477-6 17647064

[B27] Emdin SO Granstrand B Ringberg A Sandelin K Arnesson LG Nordgren H Anderson H Garmo H Holmberg L Wallgren A SweDCIS: Radiotherapy after sector resection for ductal carcinoma in situ of the breast. Results of a randomised trial in a population offered mammography screening Acta Oncol 2006 45 5 536 543 10.1080/02841860600681569 16864166

[B28] Abd El-Rehim DM Ball G Pinder SE Rakha E Paish C Robertson JF Macmillan D Blamey RW Ellis IO High-throughput protein expression analysis using tissue microarray technology of a large well-characterised series identifies biologically distinct classes of breast cancer confirming recent cDNA expression analyses Int J Cancer 2005 116 3 340 350 10.1002/ijc.21004 15818618

[B29] Tang P Wang J Bourne P Molecular classifications of breast carcinoma with similar terminology and different definitions: are they the same? Hum Pathol 2008 39 4 506 513 10.1016/j.humpath.2007.09.005 18289638

[B30] Tang P Skinner KA Hicks DG Molecular classification of breast carcinomas by immunohistochemical analysis: are we ready? Diagn Mol Pathol 2009 18 3 125 132 10.1097/PDM.0b013e31818d107b 19704256

[B31] Francis GD Jones MA Beadle GF Stein SR Bright-field in situ hybridization for HER2 gene amplification in breast cancer using tissue microarrays: correlation between chromogenic (CISH) and automated silver-enhanced (SISH) methods with patient outcome Diagn Mol Pathol 2009 18 2 88 95 10.1097/PDM.0b013e31816f6374 19430296

[B32] Shousha S Peston D Amo-Takyi B Morgan M Jasani B Evaluation of automated silver-enhanced in situ hybridization (SISH) for detection of HER2 gene amplification in breast carcinoma excision and core biopsy specimens Histopathology 2009 54 2 248 253 10.1111/j.1365-2559.2008.03185.x 19207950

[B33] Ihemelandu CU Naab TJ Mezghebe HM Makambi KH Siram SM Leffall LD DeWitty RL Frederick WA Treatment and survival outcome for molecular breast cancer subtypes in black women Ann Surg 2008 247 3 463 469 10.1097/SLA.0b013e31815d744a 18376191

[B34] Yang XR Sherman ME Rimm DL Lissowska J Brinton LA Peplonska B Hewitt SM Anderson WF Szeszenia-Dabrowska N Bardin-Mikolajczak A Differences in risk factors for breast cancer molecular subtypes in a population-based study Cancer Epidemiol Biomarkers Prev 2007 16 3 439 443 10.1158/1055-9965.EPI-06-0806 17372238

[B35] http://www.swebcg.se/index.asp?P=NatRikt

[B36] Onitilo AA Engel JM Greenlee RT Mukesh BN Breast Cancer Subtypes Based on ER/PR and Her2 Expression: Comparison of Clinicopathologic Features and Survival Clin Med Res 2009 7 1-2 4 13 10.3121/cmr.2009.825 19574486 PMC2705275

[B37] Heitz F Harter P Lueck HJ Fissler-Eckhoff A Lorenz-Salehi F Scheil-Bertram S Traut A Bois AD Triple-negative and HER2-overexpressing breast cancers exhibit an elevated risk and an earlier occurrence of cerebral metastases Eur J Cancer 2009 19643597 10.1016/j.ejca.2009.06.027

[B38] Trivers KF Lund MJ Porter PL Liff JM Flagg EW Coates RJ Eley JW The epidemiology of triple-negative breast cancer, including race Cancer Causes Control 2009 19343511 10.1007/s10552-009-9331-1 PMC4852686

[B39] Thike AA Iqbal J Cheok PY Chong AP Tse GM Tan B Tan P Wong NS Tan PH Triple Negative Breast Cancer: Outcome Correlation With Immunohistochemical Detection of Basal Markers Am J Surg Pathol 20495445 10.1097/PAS.0b013e3181e02f45

[B40] Rakha EA Elsheikh SE Aleskandarany MA Habashi HO Green AR Powe DG El-Sayed ME Benhasouna A Brunet JS Akslen LA et al Triple-negative breast cancer: distinguishing between basal and nonbasal subtypes Clin Cancer Res 2009 15 7 2302 2310 10.1158/1078-0432.CCR-08-2132 19318481

